# Effect of an Integrated Healthcare Program for Korean Rural Older Adults: A Quasi-Experimental Study

**DOI:** 10.3390/healthcare8030244

**Published:** 2020-07-30

**Authors:** Hyuk Joon Kim, Hye Young Kim, Youngran Yang, Eun Ko

**Affiliations:** 1 Research Institute of Nursing Science, College of Nursing, Jeonbuk National University, Jeonju 54896, Korea; hihikim@hanmail.net (H.J.K.); youngran13@jbnu.ac.kr (Y.Y.); 2 Department of Nursing, College of Life Science and Natural Resources, Sunchon National University, Suncheon 57922, Korea; eunko@scnu.ac.kr

**Keywords:** aged, healthcare program, physical health, mental health, social health, rural older adults

## Abstract

Studies have been conducted on the development of healthcare programs for older adults in rural areas, not only in Asia but also in Europe and the United States. However, these reports have been limited by largely non-comprehensive results, lack of demand surveys, or programs with no systematic development. The purpose of this study was to develop an integrated healthcare program for rural older adults and investigate the effects of the program. A nonequivalent control group pretest–posttest design was used. Subjects were aged over 65 and lived in the rural community. The integrated healthcare program involved 12 three-hour sessions over 12 weeks. Compared with the control group, the experimental group demonstrated significant differences in both upper extremities strengths (t = 2.74, *p* = 0.008; t = 2.03, *p* = 0.047), static balance (z = −2.38, *p* = 0.017), dynamic balance (t = −4.82, *p* < 0.001), loneliness (t = −3.02, *p* = 0.003), and role self-efficacy (t = 2.39, *p* = 0.020), but no differences for ego integration (t = 1.51, *p* = 0.137). To improve physical, mental, and social health of the rural older adults, we developed an integrated healthcare program. The program improved physical functions, loneliness, and role self-efficiency of the rural older adults. Therefore, it is recommended that healthcare professionals actively apply this program in primary healthcare institutes and elsewhere.

## 1. Introduction

The development of modern medical science, increased average life expectancy, and decreased birth rate have caused an international trend toward an aging society. The rapidly aging population has become a crucial social issue in Korea, with 14.3% of the population aged 65 years and above in 2018, expected to rise to 20.8% by 2026 [1]. This rapid aging has changed social awareness regarding older people. While awareness about older adults was previously low and involved negative attitudes towards them, there is now increasing interest in improving older adults’ physical, mental, and social health [2]. 

Older adults experience pain and dysfunction due to physical difficulties associated with aging, financial difficulties, alienation due to role loss, intergenerational conflict, loneliness, and depression [3]. A negative social atmosphere is a key factor that hinders their ego integration [4]. In rural areas, the amount of work required for agriculture has decreased due to the development of science and technology related to agricultural production. However, the agriculture-specific environment, such as working long hours in an uncomfortable position, is causing chronic agrarianism and various diseases. Additionally, the increase in sedentary lifestyles due to mechanization of farming tools has amplified the prevalence of chronic diseases such as decreased muscle mass, excessive accumulation of body fat, cardiovascular disease, and metabolic syndrome in rural older adults [5]. Comparison of the prevalence of aging-related fragility between urban and rural areas revealed that it was 10.3% in urban areas and 17.4% in rural areas, and fragile older adults are reported to be vulnerable to fatigue and declining muscle strength [6]. According to the survey of these environmental aspects, unlike in urban areas, individuals in rural areas do not undergo a life-changing processes such as retirement, and many other conditions such as facilities and programs that can provide healthcare are relatively insufficient compared to urban areas [7]. In addition, rural areas are significantly lacking in sports facilities compared to urban areas [8]. Therefore, multidirectional interventions promoting physical health and emotional satisfaction and stability are required to help rural older adults maintain integrity and perceived well-being.

According to the Roy Adaptation Model (RAM), which is widely applied in practical nursing, humans adapt to the changing environment using cognitive and controlling mechanisms, and nursing is a science that expands humans’ adaptive abilities and triggers changes in humans and the environment. Nursing also aims to assess adaptive systems, increasing individuals’ adaptability and ultimately improving health [9]. Various studies in Korea and elsewhere have described physical, emotional, and social health of specific subjects using the RAM [10]. Roy explains that as a total adaptive system, humans respond to stimuli in four adaptive modes (physiological, interdependence, role function, and self-concept modes) to maintain an integrated adaptive level. In addition, according to RAM, nursing aims to maximize health status and quality of life by improving the level of human adaptation [9]. As these four adaptive modes interact with one another and affect adaptive health outcomes, it is preferable to promote all adaptive modes in an integrated manner rather than a program focusing on only one thing [11]. Thus, to achieve the purpose of nursing, RAM can be applied in various nursing areas, and this model is considered suitable for the application in nursing intervention programs to effectively adapt to the physical, emotional, and social changes in older adults due to aging. 

### Purpose of the Study

The purpose of this study was to develop an integrated healthcare program based on the RAM and to verify its effects on the physical functions, loneliness, ego integration, and role self-efficacy of rural older adults.

## 2. Literature Review

### 2.1. Characteristics of Rural Older Adults 

Aging can be divided into physical, psychological, and socio-relational aging. In old age, physical aging is most frequently observed and the risk of cognitive disorders such as memory and intelligence decline and dementia increases [12]. Fragility and loss resulting from aging also leads to psychological changes. In particular, loneliness and depression are some of the symptoms that can occur throughout old age [13]. Even in the social sphere, older adults experience loss of role function, decrease in financial status, and isolation in human relationships. They require psychological and social re-adaptation at this time as they are often separated from their social circles due to physical limitations, retirement, or loss due to the death of their spouse [13]. Moreover, older adults are less likely to participate in the community, and thus they spend most of their time in a single type of leisure activity and are less likely to participate in social activities or meetings with neighbors or friends [14,15].

In particular, older adults living in rural areas were found to have living conditions and welfare needs that are different from those of the older adults living in urban areas. In the case of Korea, the gap between regions in terms of living environment and population characteristics between urban and rural areas has widened along with economic development. Compared to cities with better access to welfare services, rural areas have a negative impact on the health status and quality of life of rural older adults due to the aging population, labor shortage, and lack of preventive healthcare services and facilities [16]. These social and economic imbalances in urban and rural areas cause various health-related problems, such as poor overall health condition, weak mental health, and declining social network in older adults in rural areas. This warrants more attention in terms of the healthcare of older adults in rural areas [17].

### 2.2. Health Care Programs for Older Adults 

The World Health Organization defines health as a state of physical, psychological, and social well-being. Therefore, for healthcare of older adults, programs aimed at improving psychological and social health and physical aspects should be planned. Deterioration in physical function due to aging leads to decreased immunity, increased vulnerability to disease, and reduced recovery, and thus training to improve physical health is needed [7,18,19]. Healthcare programs including physical activity not only help maintain physical health by increasing brain blood flow, such as preventing dementia and improving cognition, strengthening cardiovascular functions, and preventing bone density, but also have a positive effect on psychological well-being, such as life satisfaction, loneliness, and depression [20].

Therefore, healthcare programs for older adults mostly include physical activities as a major component. Since the National Blueprint in 2001, the USA has been striving to promote physical activity for older adults in many ways. In particular, experts are beginning to pay attention to the verification of the effectiveness of physical activity programs, and these verified programs are being disseminated systematically. Major healthcare programs in the USA that are provided to senior citizens include Active Choices, Strong for Life, Healthy Moves for Aging Well, Tai Chi, and Fit and Strong, among others, which are reported to be effective in preventing falls and improving independence and reducing depression among older people [21]. On the basis of the “Fit für 100” program produced by the German National Sport University, Germany offers exercise programs for those aged 80 years or older as its main targets [21]. In addition, Japan provides exercise programs for weak and older adults with impaired cognitive skills as a measure to prevent the need for long-term healthcare in 2011, offering around 17 exercise programs for individuals and groups [21]. 

Although prior studies on health management programs for rural older adults focused on disease, loneliness, and poverty [2], they did not examine the multifaceted nature of the older adults and focused instead on single programs such as exercise [21,22,23], music [24,25], art [26], horticultural therapy [27], and laughter therapy [15]. Furthermore, although these programs may reduce cortisol [27] and improve physical function [21,22,23,27], cognitive function [23], and depressive disorders [21,26], they were restricted to analyzing the pre- and posttests of the experimental group [28], and did not investigate the subjects’ needs [23,26,28] or develop a systematic program [10,27]. 

In order to lead a healthy life in old age, one must adapt well to the changes and problems in various aspects that emerge in old age. Integrated programs are needed to maintain physical and social health through active social participation and mental health that can reduce anxiety and fear of isolation or death, alienation, and helplessness [2,12,13]. To date, however, research on the physical, mental, and social aspects of older adults has been rare, with most involving single interventions such as exercise-oriented approaches, music, and art therapy [21,23,24,25,26,27]. In Korea, as the older adult population is growing faster than in any other country worldwide, development and objective evaluation of integrated healthcare programs are required to consider factors such as improving the cognitive and physical abilities, maintaining positive interpersonal relationships through collective activities and promoting happiness, establishing life goals, and expanding role functions through personal growth.

## 3. Materials and Methods 

### 3.1. Study Design

A nonequivalent control group pretest–posttest study design was used.

### 3.2. Participants and Setting

A convenience sample was recruited from J province in South Korea. The inclusion criteria were (1) being a rural community-residing older adult aged 65 and over, (2) being able to communicate, and (3) the ability to understand the study purpose and provide written consent to voluntarily participate. The following were excluded from this study: (1) individuals who have participated in any health-related program within one year, (2) being unable to participate in physical activities due to movement difficulties, and (3) being diagnosed with serious diseases such as Alzheimer’s or cancer. 

**Figure 1:**
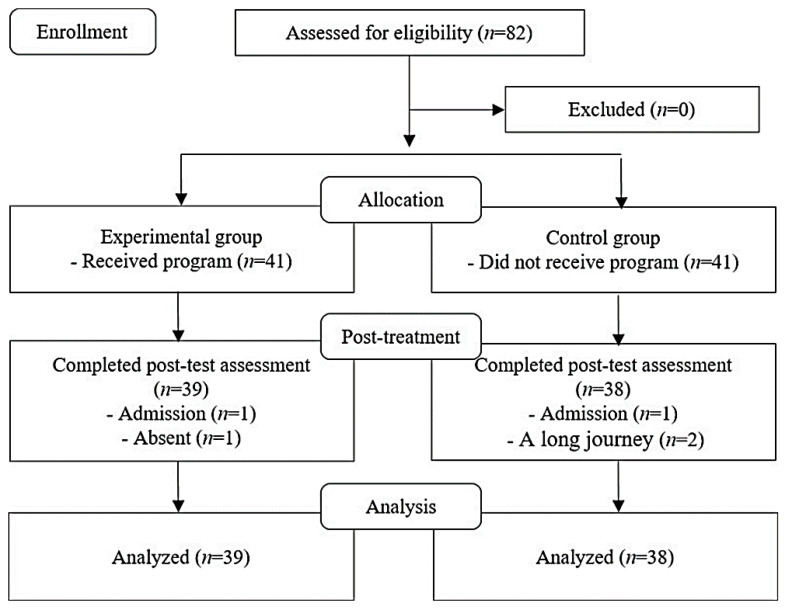
Flow diagram in this study.

**Figure 2:**
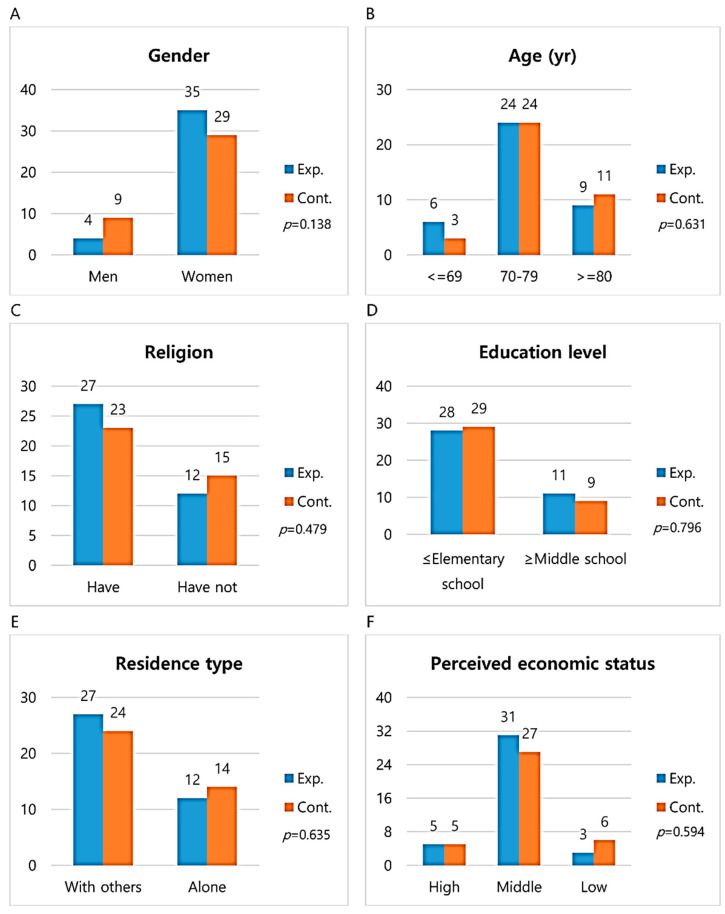
Homogeneity of general characteristics between two groups. (**a**) No significant difference in gender (χ^2^ = 2.47). (**b**) No significance difference in age (χ^2^ = 1.18). (**c**) No significance difference in religion (χ^2^ = 0.64). (**d**) No significance difference in education level (χ^2^ = 0.21). (**e**) No significance difference in residence type (χ^2^ = 0.32). (**f**) No significance difference in perceived economic status (χ^2^ = 1.26). Significance level was set to 0.05. Exp. = Experimental group; Cont. = Control group.

The experimental group sample (*n* = 41) was recruited from two halls for the older adults in J province, while the control group sample (*n* = 41) was convenience sampled from J province and had similar general characteristics and living environments but no social intercourse with the experimental group, giving a total of 82 subjects. During the study, two subjects from the experimental group (one admission, one absent for two sessions) and three from the control group (one admission, two on long journeys) dropped out, resulting in a final analysis of 77 subjects (experimental group = 39; control group = 38) (Figure 1). The average age of the experimental group was 74.7 ± 5.26 and the average age of the control group was 76.6 ± 5.52. The results of the homogeneity test of the general characteristics of the subject are shown in Figure 2. There was no significant difference in gender (χ^2^ = 2.47, *p* = 0.138), age (χ^2^ = 1.18, *p* = 0.631), religion (χ^2^ = 0.64, *p* = 0.479), education level (χ^2^ = 0.21, *p* = 0.796), residence type (χ^2^ = 0.32, *p* = 0.635), and perceived economic status (χ^2^ = 1.26, *p* = 0.594) between the control and experimental groups (Figure 2). 

This study was certified by the ethics review board (IRB no. 2017-06-015-002). All subjects provided written informed consent to voluntarily participate and were given small gifts. After the study, the control group subjects were given workbooks from the integrated healthcare program.

### 3.3. Procedure

#### 3.3.1. Development of an Integrated Healthcare Program for the Rural Older Adults 

The program was developed on the basis of a needs analysis of the subjects and existing literature to investigate effective contents for integrated healthcare for the older adults. The program was developed in the order of goal-setting, program construction, and publishing workbooks. 

To construct the program contents, we examined previous literature from the past 10 years with the key words “rural older adults health”, “rural older adults programs”, “integrated healthcare program”, and “integrated health promotion program”. As a result of reviewing data from papers we searched for that were written over the past 10 years, the most frequently reported effects were found through providing exercise-related programs for physical health management.

A semi-structured needs analysis survey was then conducted with 20 rural older adults who visited a center for the older adults in J province and who could understand the survey purpose and who provided written consent to voluntarily participate. Six respondents (30.0%) had participated in health-related programs. When asked about difficulties in their daily lives, health-related difficulties were the most common, followed by loneliness, economic problems, and communication in personal relationships. The most sought-after program was “how to prevent Alzheimer’s disease”, followed by “how to be healthy”, “home exercise”, “singing”, “dancing”, and “drawing”. According to the needs analysis, the program included contents on physical activities, recreation, strengthening cognitive abilities, and communication for good personal relationships, among others. 

Increasing physical functions, ego integration, role self-efficacy, and decreasing loneliness were selected as the program goals to increase the physical, mental, and social health of the older adults. 

Finally, the RAM was selected as the theoretical basis of the program to describe the successful adaptation outcome of the rural older adults. On the basis of prior studies, the program was divided into 12 sessions. To verify the program’s content validity, we selected an expert group of four people—three nursing professors and one program staff member of the health center—who assessed the validity of each program topic on a four-point Likert scale. The Content Validity Index (CVI) of all topics exceeded 0.75, determining content validity. The developed program was named the “Integrated Healthcare Program for the Older Adults”.

Each session was designed to comprehensively connect the four adaptive modes from the RAM to increase physical functions, ego integration, and role self-efficacy and decrease loneliness. First, to improve the physical function of the subjects, which was considered the physiological mode, we included lectures on topics such as “successful aging”, “hypertension and diabetes self-management”, “first aid in daily life”, “safety management in older adults”, “nutrition and health”, and “oral care in old age” in the program contents. It also included cognitive strengthening exercise and small-group activities at each session to improve physical functions. Second, to enhance the ego integration, which is regarded as the self-concept mode, we included lectures on “what is logical function?” and activities such as “recall past and candle ceremony”, “create a proposal card/praise others”, and “speaking about feelings regarding the program”. Third, to increase role self-efficacy, which was considered as the role function mode, we included small-group activities such as team building, cognitive puzzle (group), cognitive board games (group), and cooking practices. Finally, to decrease loneliness, which was regarded as the interdependence mode, we delivered lectures on “communication skills in neighbors” and small-group activities such as team building, expressing through colors (group), cognitive puzzle (group), laughing, singing, and dancing, among others. The specific programs are shown in Table 1.

#### 3.3.2. Intervention

##### Pretest

Before the study intervention, the authors and experts (three nursing professors who are certified Silver Cognitive Play Instructors, one oriental medicine professor, one dentist, and two laughter therapists) were consulted regarding the study and program purpose. One-hour training was also provided to two study assistants on the study purpose and how to measure the physical functions. Afterwards, the evaluators’ consistency was calculated with four standard subjects using the Intraclass Correlation Coefficient (ICC), wherein an inter-evaluator consistency of 0.90 was achieved.

Data collection was conducted at a hall for the older adults in J province for the experimental group and control group. Surveys were conducted as a single blind study, either by allowing the subjects to complete the survey by themselves or by conducting face-to-face interviews. The physical functions were measured by two assistants.

##### Intervention

The intervention was conducted from September to December 2017 in the seminar room of a hall for the older adults in J province. To ensure all subjects had identical programs, all participants attended the program simultaneously as a single group. Each session was managed by the first author and seven experts according to the program contents. The authors phoned or texted subjects the day before to remind them about the program. To encourage participation, there was an attendance board on the wall where subjects could place stickers and subjects with excellent attendance were rewarded. The average program attendance rate after 12 weeks was 99.6%.

##### Posttest

The posttest was conducted directly after the program by two assistants in the same manner as the pretest.

### 3.4. Data Collection and Outcome Measures

Data collection was conducted from September to December 2017. Approximately 70 to 80 min was taken to conduct surveys and measure physical functions. Identical pretests were conducted on the control and experimental groups, and the posttest was conducted after 12 weeks of the program.

Physical functions were measured according to Rikli and Jones’ [29] Senior Fitness Test (SFT) Manual. Left and right upper extremity strength was measured using a dynamometer (Takei, Niigata city, Japan), recording the average for each 0.1 kg twice, for left and right sides. Static balance was measured per second standing on one foot with open eyes, and the better figure out of two tries was used. Dynamic balance was measured using the 8-feet Up-and-Go Test by Mathias et al. [30]. The lower the measurement, the better the dynamic balance. 

Loneliness was measured using the University of California, Los Angeles (UCLA) Loneliness Scale, developed by Russell [31] and validated in Korean by Kim [32]. This tool comprises 20 questions, and higher scores on a four-point Likert scale show higher loneliness. Regarding the reliability of the tool, the Cronbach’s α was 0.93 in Kim [32] and 0.70 in this study. 

Ego integration was measured using Hong’s [33] Ego Integration Scale, comprising 16 questions measured on a five-point Likert scale (higher scores show higher ego integration). Regarding tool reliability, Cronbach’s α was 0.83 at the time of development, and 0.71 in this study. 

Role self-efficacy was measured using the 13 questions on role self-efficacy from Kim and Shin’s [34] Successful Aging Scale. Higher scores on the five-point Likert scale demonstrate higher role self-efficacy. Regarding tool reliability, Cronbach’s α was 0.94 at the time of development, and 0.83 in this study. 

### 3.5. Data Analysis

The data were analyzed using SPSS version 26.0 (IBM Corp., Armonk, NY, USA) at a significance level of 0.05. Independent *t*-test and Mann–Whitney *U* test were used for the continuous variables, and Fisher’s exact test was used to analyze cases when the minimum expected frequency was below 5 for over 20% of the total cells during the χ^2^ test for the categorical variables of the homogeneity test of general characteristics and prior dependent variables. The Shapiro–Wilk test was used to examine the normality of dependent variables. Nonparametric test was used for static balance since there was no normal distribution. The data were analyzed by using an independent t-test and Mann–Whitney *U* test to identify the differences between the experimental group and the control group at the end of the program.

## 4. Results and Discussion

### 4.1. Homogeneity of Dependent Variables between Groups

**Figure 3:**
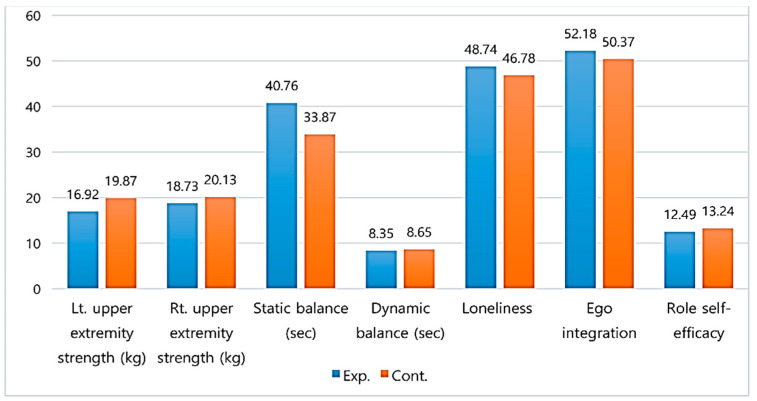
Homogeneity of dependent variables between two groups. Data represent the mean or median values. Exp. = Experimental group; Cont. = Control group; Lt. = Left; Rt. = Right.

There were no significant differences between the two groups in left upper extremity strength (t = −1.99, *p* = 0.051), right upper extremity strength (t = −1.56, *p* = 0.123), static balance (z = −1.38, *p* = 0.085), dynamic balance (t = −0.66, *p* = 0.509), loneliness (t = 1.82, *p* = 0.072), ego integration (t = 1.50, *p* = 0.137), and role self-efficacy (t = −1.13, *p* = 0.260) (Figure 3).

### 4.2. Effect of the Integrated Healthcare Program

**Figure 4:**
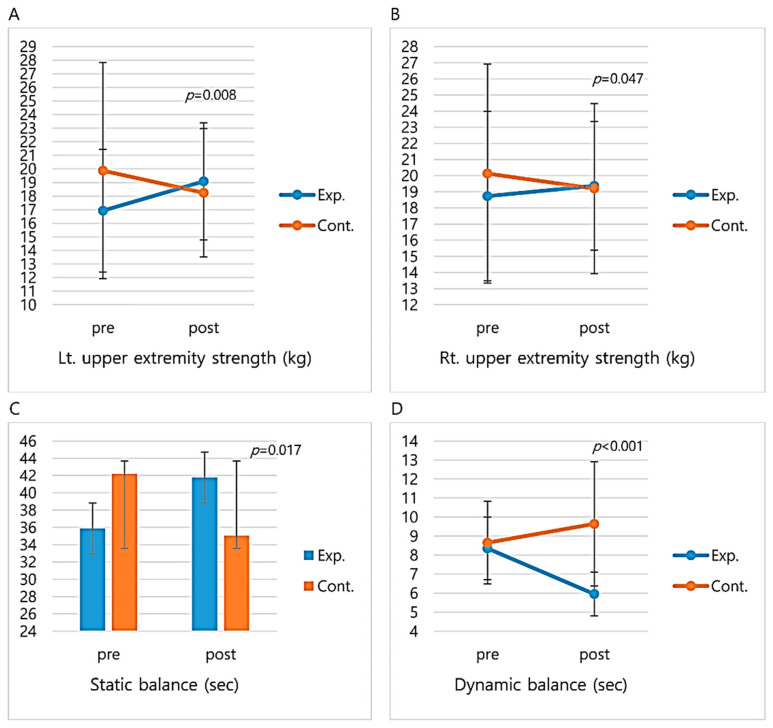
Comparison of physical functions before and after the integration healthcare program. (**a**) Change in Lt. upper extremity strength mean score. (**b**) Change in Rt. upper extremity strength mean score. (**c**) Change in static balance median score. (**d**) Change in dynamic balance mean score. Data represent the mean or median values, with standard deviation or interquartile range. Significance level was set to 0.05. Exp. = Experimental group; Cont. = Control group; Lt. = Left; Rt. = Right.

**Figure 5:**
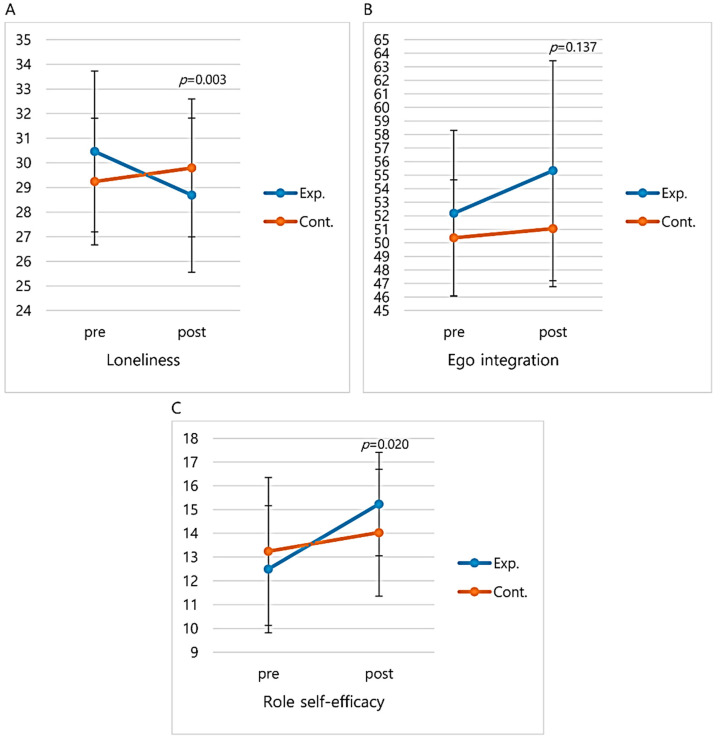
Comparison of loneliness, ego integration, and role self-efficacy before and after the integration healthcare program. (**a**) Change in loneliness mean score. (**b**) Change in ego integration mean score. (**c**) Change in role self-efficacy mean score. Data represent the mean values with standard deviation. Significance level was set to 0.05. Exp. = Experimental group; Cont. = Control group.

The effects of the integrated healthcare program are shown in Figure 4 and Figure 5. First, there was a meaningful difference (t = 2.74, *p* = 0.008) in the left upper extremity strength of the two groups, with the experimental group increasing from 16.92 ± 4.52 kg before the program to 19.08 ± 4.31 kg after, while the control group decreased from 19.87 ± 7.95 kg before the program to 18.24 ± 4.72 kg after. There was a meaningful difference (t = 2.03, *p* = 0.047) in the right upper extremity strength of the two groups, with the experimental group increasing from 18.08 ± 4.47 kg before the program to 19.37 ± 3.99 kg after, while the control group decreased from 20.13 ± 6.79 kg before the program to 19.20 ± 5.27 kg after. There was a statistically meaningful difference (z = −2.38, *p* = 0.017) in the static balance of the two groups, with the experimental group increasing from 35.88 before the program to 42.20 after, while the control group decreased from 41.78 before the program to 35.04 after. There was a meaningful difference (t = −4.82, *p* < 0.001) in the dynamic balance of the two groups, with the experimental group decreasing from 8.35 ± 1.64 s before the program to 5.95 ± 1.15 s after, while the control group increased from 8.65 ± 2.17 s before the program to 9.64 ± 3.27 s after (Figure 4). 

After the integrated healthcare program, the right upper extremity strength, left upper extremity strength, static balance, and dynamic balance demonstrated meaningful difference. These results are identical to Nishiguchi et al. [18], who found an increase in muscle strength following a 12-week integrated healthcare program including a low-intensity exercise program for the older adults in senior centers. They differ, however, from Marmeleira et al. [23], where an integrated eight-session healthcare program for the female older adults did not improve muscle strength. Although the effects of exercise vary depending on type and duration, a strategy is required that allows the older adults to increase muscle strength in their daily lives, as increased muscle strength in the upper and lower bodies greatly benefits their ability to lead stable and independent lives [6,22].

The results also demonstrated a meaningfully higher static and dynamic balance of the experimental group than the control group. Similar results were found by Wongcharoen et al. [35], where balance rapidly increased after a 12-week seniorobics program, and by Oliveira Goncalves et al. [36], where 16-week exercise programs customized for fall prevention were shown to be effective. Static balance is the basic ability to maintain a stable center of the body and is a requirement for a safe everyday life [36]. Balance substantially contributes to securing the safety of the older adults in their everyday lives, and needs to be trained through exercise to prevent injury from falls, etc. [37,38]. In this study, silver exercises and cognitive walking composed of simple repetitive movements at a moderate intensity level and coupled with music were developed to increase physical functions including muscle strength. Accurate beats and appropriate activity speed are believed to enable stable switches of movement, and movements that lasted for two to four beats particularly helped to improve balance.

Second, in this study, there was a meaningful difference (t = −3.02, *p* = 0.003) in the loneliness of the two groups, with the experimental group decreasing from 30.46 ± 3.27 before the program to 28.69 ± 3.13 after, while the control group increased from 29.24 ± 2.57 before the program to 29.79 ± 2.80 after (Figure 5). Thus, the experimental group participants demonstrated a meaningful decrease in loneliness, supporting studies on various leisure activities and programs in Korea [14,38]. The program decreased older adults’ loneliness by providing the opportunity to socialize and relieve stress, and encouraging positive attitudes through laughter therapy, games, music, and art, among others. Liu et al. [13] analyzed the factors affecting older adults’ loneliness and demonstrated that support from children does not help much due to generation gap differences and lack of sympathy; instead, it is support from friends that relieves loneliness. In this program, loneliness decreased with group participation, allowing for the formation of natural friendships and meaningful relationships through team activities and mutual cooperation. Therefore, it is suggested that small-group or team activities be strengthened when implementing healthcare programs for older adults. 

Third, in the current study, there was a meaningful difference (t = 2.39, *p* = 0.020) in the role self-efficacy of the two groups, with the experimental group increasing from 12.49 ± 2.67 before the program to 15.23 ± 2.18 after, while the control group increased from 13.24 ± 3.11 before the program to 14.03 ± 2.67 after (Figure 5). These results demonstrated that the role self-efficacy of the older adults increased after participating in the program. During the program, the older adults were given classes on effective communication with family, friends, and neighbors; first aid; and role as a silver leader to increase their role functions within and outside of their families. Results from various studies demonstrate that volunteer activities increase older adults’ self-efficacy [2,39], while participation in health promotion programs [40], customized cardiopulmonary resuscitation programs [41], and leisure activities [42] increased role self-efficacy of the older adults’ physical or social activities and relationship formation, which align with our results. Losing physical abilities as older adults does not necessarily impair psychosocial functions; instead, self-efficacy can be maintained through knowledge or expertise, which compensate for the loss of physical abilities. Furthermore, as many studies have demonstrated, the higher the self-efficiency, the better it can adapt to aging [2,4,43], and increasing self-efficacy through active social participation and self-management should be an essential component of older adult intervention programs.

However, in this study, there was no meaningful difference (t = 1.51, *p* = 0.137) in the ego integration of the two groups, with the experimental group increasing from 52.18 ± 6.12 before the program to 55.33 ± 8.12 after, while the control group increased from 50.37 ± 4.28 before the program to 51.05 ± 4.28 after (Figure 5). This result was contrary to Li et al.’s study [44] showing that close relationships with neighbors and friends within the community compensate for psychological maladaptation due to role loss, being a crucial factor for ego integration of older adults. In this study, the program was developed to include activities such as reminiscence about the past and picturing the future, and positive acceptance of past and future through candle ceremonies to increase ego integration suggested in the studies by Ciasca et al. [26] and Jo and Song [38]. These results seem to be insufficient to improve the ego integration as our research deals with various programs. That is, unlike the exercise program, activities to improve ego integration consist of 3 sessions out of 12 sessions, and it seems that ego integration was not improved in this study. It is also believed that the ego integration does not change easily. 

In this study, a conceptual framework was established through RAM in which subjects adapt through four types of adaptive modes: physical function, ego integration, role self-efficacy, and loneliness. Therein, the results of this study showed that the integrated healthcare program improved the static and dynamic balance among the physical functions of the elderly individuals; increased their ego integration and role self-efficacy; decreased their loneliness; and resulted in the improvement of physical, social, and mental health. The results of this study can be seen as supporting previous studies that demonstrated improved emotional health and self-efficacy through physical activities for the elderly adults with disabilities on the basis of RAM [45], and improved emotional health by reducing loneliness and depression through social support [46].

The limitation of this study is that the subjects were selected from one specific area using the convenience sampling method, leading to concerns about generalizing the study results. Furthermore, the physical functions of the older adults were only evaluated by measuring the upper extremity strength, static balance, and dynamic balance, leading to limitations in explaining the overall physical functions of the older adults. 

## 5. Conclusions

Studies have been conducted on the development of healthcare programs for rural older adults, not only in Asia but also in Europe and the United States. However, previous reports were restricted to analyzing the pre- and posttests of the experimental group and did not investigate older adults’ needs or develop a systematic program. This study attempted to develop an integrated healthcare program for the older adults on the basis of RAM and to verify its effectiveness. The integrated healthcare program developed in this study comprises an integrated program to improve the physical functions, role self-efficacy, ego integration, and loneliness of rural older adults. 

The study subjects were senior citizens aged 65 or older, with 39 experimental groups participating in the program for 12 weeks, at 120 min per week; 38 control groups did not receive the integrated healthcare program. Measurement tools for verifying the effectiveness of the program were Senior Fitness Test, UCLA Loneliness Scale, Eco Integration Scale, and Successful Aging Scale. The data analysis was performed using SPSS version 26.0 (IBM Corp., Armonk, NY, USA), and differences in dependent variables between the two groups were identified using the independent *t*-test and Mann–Whitney *U* test. As a result of this study, the experimental group participating in the Integrated Healthcare Program had statistically significant improvements in all four types—left upper extremity strength (t = 2.74, *p* = 0.008), right upper extremity strength (t = 2.03, *p* = 0.047), static balance (z = −2.38, *p* = 0.017), and dynamic balance (t = −4.82, *p* < 0.001)—compared to the control group. Moreover, loneliness (t = −3.02, *p* = 0.003) and role self-efficacy (t = 2.39, *p* = 0.020) improved statistically significantly compared to the control group. The results of this study supported the previous studies [2,14,18,35,36,38,39]. However, ego integration score improved after the program, but there was no significant difference between the experimental and control groups. In this study, activities to promote ego integration were organized into 3 of the 12 sessions. Therefore, further studies need to organize programs with more diverse activities and longer periods to improve ego integration. 

In conclusion, this study demonstrated that the integrated healthcare program we developed is effective in increasing some physical functions and role self-efficacy and decreasing loneliness. The program’s success was due to the fact that the core strategy involves providing various activities that cater to rural older adults to increase participation and interest. This study is meaningful in confirming the role of a healthcare program that integrates physical, mental, and social health factors. Furthermore, to prepare for an aging society, programs facilitating a healthy and satisfactory life after retirement by successfully adapting to the physical, psychological, and social changes that accompany aging should be provided as ongoing rather than one-time programs. Therefore, it is recommended that healthcare professionals actively apply this program in primary healthcare institutes and elsewhere, with public nurses serving either as the main program operators or cooperating with other healthcare personnel.

## Figures and Tables

**Table 1:** Integrated healthcare program for older adults based on the Roy Adaptation Model.

Session	Contents	Rationale Based on Roy Adaptation Model	Time(min)
1	▹ Entrance ceremony/program introduction ▹ Write a participation pledge ▹ Pre-test		30 10 80
2	▹ Lecture: Successful aging ▹ Exercise: Silver cognitive strengthening exercise ▹ Activity: Self-introduction, team building, and game	▹ Physiologic mode ▹ Interdependence mode ▹ Role function mode	30 20 70
3	▹ Lecture: Communication skill in neighbors ▹ Exercise: Silver cognitive strengthening exercise	▹ Interdependence mode ▹ Physiologic mode	90 30
4	▹ Lecture: What is cognitive function? ▹ Exercise: Silver cognitive strengthening exercise ▹ Activity: Recall past and candle ceremony	▹ Self-concept mode ▹ Physiologic mode	30 20 70
5	▹ Lecture and practice: Hypertension and diabetes self-management ▹ Exercise: Silver cognitive strengthening exercise ▹ Activity: Expressing through colors (group)	▹ Physiologic mode ▹ Interdependence mode	50 20 50
6	▹ Exercise: Silver cognitive strengthening exercise ▹ Activity: Laughter therapy	▹ Physiologic mode ▹ Interdependence mode	100 20
7	▹ Lecture: First aid in daily life ▹ Exercise: Cognitive strengthening step ▹ Activity: Cognitive puzzle game (group)	▹ Physiologic mode ▹ Role function mode ▹ Interdependence mode	50 20 50
8	▹ Exercise: Line dance ▹ Activity: Singing and dancing	▹ Physiologic mode ▹ Interdependence mode	60 60
9	▹ Lecture: Safety management in older adults ▹ Exercise: Cognitive strengthening step ▹ Activity: Cognition board games (group)	▹ Physiologic mode ▹ Role function mode ▹ Interdependence mode	30 20 70
10	▹ Lecture: Nutrition and health ▹ Exercise: Silver cognitive strengthening exercise ▹ Activity: Cooking practice	▹ Physiologic mode ▹ Interdependence mode ▹ Role function mode	30 20 70
11	▹ Lecture: Oral care of old age ▹ Exercise: Cognitive strengthening step ▹ Activity: Create a praise card/praise others	▹ Physiologic mode ▹ Self-concept mode	30 20 70
12	▹ Activity: Speaking about feelings regarding the program ▹ Awards ceremony ▹ Post-test	▹ Self-concept mode ▹ Interdependence mode	30 20 70
